# Cross-Culture Validation of the HIV/AIDS Stress Scale: The Development of a Revised Chinese Version

**DOI:** 10.1371/journal.pone.0152990

**Published:** 2016-04-04

**Authors:** Lu Niu, Yangyang Qiu, Dan Luo, Xi Chen, Min Wang, Kenneth I. Pakenham, Xixing Zhang, Zhulin Huang, Shuiyuan Xiao

**Affiliations:** 1 Department of Social Medicine and Health Management, School of Public Health, Central South University, Changsha, Hunan, China; 2 Hunan provincial Center for Disease Control and Prevention, Changsha, Hunan, China; 3 The First Hospital of Changsha, Changsha, Hunan, China; 4 School of Psychology, University of Queensland, Brisbane, Australia; 5 Changsha Center for Disease Control and Prevention, Changsha, Hunan, China; UCSF, UNITED STATES

## Abstract

**Background:**

Being HIV-infected is a stressful experience for many individuals. To assess HIV-related stress in the Chinese context, a measure with satisfied psychometric properties is yet underdeveloped. This study aimed to examine the psychometric characteristics of a simplified Chinese version of the HIV/AIDS Stress Scale (SS-HIV) among people living with HIV/AIDS in central China.

**Method:**

A total of 667 people living with HIV (92% were male) were recruited from March 1^st^ 2014 to August 31^th^ 2015 by consecutive sampling. A standard questionnaire package containing the Chinese HIV/AIDS Stress Scale (CSS-HIV), the Chinese Patient Health Questionnaire-9 (PHQ-9), and the Chinese Generalized Anxiety Disorder Scale (GAD-7) were administered to all participants, and 38 of the participants were selected randomly to be re-tested in four weeks after the initial testing.

**Results:**

Our data supported that a revised 17-item CSS-HIV had adequate psychometric properties. It consisted of 3 factors: emotional stress (6 items), social stress (6 items) and instrumental stress (5 items). The overall Cronbach’s α was 0.906, and the test-retest reliability coefficient was 0.832. The revised CSS-HIV was significantly correlated with the number of HIV-related symptoms, as well as scores on the PHQ-9 and GAD-7, indicating acceptable concurrent validity.

**Conclusion:**

The 17-item Chinese version of the SS-HIV has potential research and clinical utility in identifying important stressors among the Chinese HIV-infected population and in understanding the effects of stress on adjustment to HIV.

## Introduction

China’s HIV/AIDS prevention strategies and treatment measures have been implemented with notable effectiveness, but the number of people living with HIV as well as new infections per year continues to increase [1]. Nationally, as many as 497,000 HIV-infected people registered on the China Information System for Disease Control and Prevention by the end of October 2014 [2]; however, an expert panel estimated that the actual number was 780,000 by the end of 2011[3]. Case reporting data shows that the number of newly diagnosed has increased each year from 20,450 in 2011[4] to 45,145 in 2014 [5]. According to the *2014 China AIDS response progress report*, among newly HIV-infected cases, sexual transmission, has become the primary mode of transmission, increasing from 33.1% in 2006 to 90.8% in 2013[1]. Notably, the proportion of people infected through homosexual transmission has increased significantly from 2.5% in 2006 to 21.4% in 2013[1].

Being infected with HIV is an extremely stressful experience for many individuals, which affects almost every aspect of a person’s life [6, 7]. A number of studies show that higher stress among people living with HIV is related to greater psychological morbidity, such as depression and anxiety [8]. Stress has also been documented to elevate risks of health-damaging behaviors, like smoking [9], alcohol use [10], and unsafe sex [11]. All of these factors eventually lead to lower quality of life and worse medical outcomes for people living with HIV [12]. AIDS is currently a manageable chronic infectious disease, and how to effectively and efficiently provide HIV care in medical facilities and communities is increasingly recognized as a crucial public health issue. Assessing stress experienced by people living with HIV is, therefore, an important first step in understanding adjustment to HIV and in assisting with psychosocial supports, such as stress management strategies.

With different operationalizations of the construct of stress, several established generic instruments or modified versions of these inventories (e.g., the Life Event Scale [13] and the Perceived Stress Scale [6, 14–16]) have been used to investigate stress among people living with HIV. These generic scales are not likely to be sensitive to stress within the HIV context, hence several HIV-specific life-event scales have been developed [6, 17–20]; however, there are no validation data for these scales. Amongst the HIV-specific stress measures for people living with HIV, the Perceived Stress Scale for People Living with HIV/AIDS [21] is the only validated Chinese stress scale. However, it consists of eight factors, some of which have conceptual overlap. For example, sexual relationship, social acceptance/rejection, and work related issues could all be categorized into social/psychological problems conceptually.

In 2002 Pakenham and Rinaldis developed a measure of stress specific to HIV/AIDS called the HIV/AIDS Stress Scale (SS-HIV) [22]. Pakenham and Rinaldis [22] used Lazarus and Folkman’s [23] conceptualization of stress and coping to guide the development of the SS-HIV. This conceptualization defines stress as “a relationship between the person and the environment that is appraised by the person as taxing or exceeding his or her resources and endangering his or her well-being” [23]. According to this definition, one of the key elements of the stress process is a person’s appraisal of the stressfulness of an event. Hence, the SS-HIV gauges respondent’s subjective appraisal of the stressfulness of HIV/AIDS-specific stressors. Furthermore, Pakenham and Rinaldis [22] showed that the SS-HIV was related to appraisal processes and other theoretical constructs in Lazarus and Folkman’s conceptualization of stress and coping. The initial SS-HIV composed of 29 items was developed via qualitative interviews with 96 homosexual/bisexual HIV-positive men in Australia [22, 24]. Each item represents one category of HIV/AIDS related problems that people living with HIV experienced in the preceding month, and two example problems were provided for each item using the qualitative data gained from earlier research [22, 24]. By exploratory factor analysis, a 23-item SS-HIV with three factors was generated; the three factors were social stress, instrumental stress and emotional/existential stress [22]. The 23-item SS-HIV has shown good test-retest reliability, internal consistency and concurrent validity in the gay/bisexual HIV-infected population [22]. In this study, the SS-HIV was adopted to measure stress for Chinese people living with HIV.

Illness is experienced within a cultural and social context which, in turn, can shape how people appraise aspects of their illness and how they cope with it [25]. Hence, the meaning of items in a self-report scale that measures illness-related stress may vary from one cultural and social context to another [26]. This is particularly likely in relation to HIV given the associated cultural and social factors (e.g., sexual orientation, discrimination, stigma, and diagnosis concealment) [27]. Cross-cultural research that examines the reliability and validity of illness-related self-report scales is important to determine whether a remapping of the underlying meanings of scale factors or items occurs when a measure is used in a different culture [28]. In the present study we explore the extent to which the underlying meanings of factors and items in the SS-HIV change from a Western to a Chinese cultural and social context.

In 2003 Lin et al. [27, 29] translated the SS-HIV into Chinese and applied it to HIV-positive former plasma/blood donors in Henan province, China. Then in 2007, Gao et al [30] also used this instrument among people living with HIV recruited from multiple settings in Guangzhou without clearly stating the translation and validation process. Both studies reported acceptable internal reliability coefficients for the Chinese versions of the SS-HIV (Cronbach’s α ranged from 0.55 to 0.75) [29, 30], but the translations used in these Chinese versions vary. Additionally, other psychometric characteristics of these Chinese versions of the SS-HIV, such as test-retest reliability and validity, have not been examined. Hence, there is as yet no standardized and validated Chinese version of the SS-HIV.

In this study, we aim to develop a culturally adapted and validated Chinese HIV-related Stress Scale (CSS-HIV), which would help to elucidate stress processes involved in HIV diagnosis, management, and treatment among people living with HIV in China. We also aim to explore the applicability of CSS-HIV among Chinese HIV-infected people, and shed light on its psychometric properties.

## Methods

### Ethics Statement

Ethics approval was obtained through the Human Research Ethics Committee of Central South University, and written informed consent has been obtained from all participants.

### Participants and procedures

We used a cross-sectional design with consecutive sampling. Changsha City is the provincial capital of Hunan province in China. By the end of 2014, Hunan Province had 23,515 cumulated diagnosed HIV/AIDS cases, including 6,847 deceased cases, which represents the 7th highest number of cases of HIV/AIDS among all provincial districts in China. Sexual contact has become the primary mode of transmission in China, with 96% of new infections in Hunan province in 2014 being infected by sexual contact [31].

During March 1^st^ 2013 to August 31^th^ 2014 a total of 667 people living with HIV who attended the free voluntary counseling and testing clinic of Changsha CDC and the Changsha Infectious Disease Hospital were recruited. Participants had to be aged ≥18 years old and living in Changsha City, including 6 districts and 3 counties. The response rate was 95.3% (667/700). All participants signed the informed consent form, and completed a questionnaire package. Thirty-eight participants were selected randomly to complete the questionnaire again in four weeks after the initial testing. The sampling procedure of this study has been described in detail elsewhere [32].

### Measurements

#### Demographics and HIV information

Demographics such as gender, age, educational background, employment, and marriage status were collected by a purpose-built questionnaire. Medical and HIV related information was obtained from records in the HIV/AIDS registered management system, including mode of HIV-transmission, time since HIV-infection, CD4 count (/μl), and number of HIV-related symptoms as follows: persistent fevers for over 1 month; persistent diarrhea or watery stools for over 1 month; persistent cough for over 1 month; over 10% weight loss in the last 3 months; white patches in the mouth (thrush); recurrent herpes simplex; active tuberculosis; and others (indicated by participants).

#### Chinese HIV/AIDS stress (CSS-HIV)

The 23-item SS-HIV is a measure of stress specific to HIV/AIDS [22]. It consists of three subscales: (1) social stress (10 items), which includes stressful social events such as isolation, stigma, difficulties on disclosure of HIV status and interpersonal relationships; (2) instrumental stress (6 items), which includes daily practical difficulties associated with HIV/AIDS related financial, transport, and treatment problems; and (3) emotional/existential stress (7 items) including HIV/AIDS related grief/bereavement, distressing emotions, concerns about death, and religious issues. Each item represents one problem category with two related examples. For instance, the item *Distressing emotions related to HIV/AIDS* also lists two examples, *you feel angry or fearful; you feel anxious or depressed* (see S1 Appendix). Respondents rate their perception of how distressing each problem item is on a 5-point Likert scale that ranges from 0 (not at all) to 4 (extremely), with higher scores indicating higher levels of stress.

Approved by the first author of the SS-HIV (Pakenham [22]), a panel of three bilingual public health professors, who were also trained in psychiatric or psychological fields, translated the original English version of the SS-HIV into simplified Chinese. Several meetings were held to review and discuss the difficulties encountered by individual translators during the translation process. The translator also had to seek clarification from the first author of the SS-HIV regarding the meaning of several items. Another meeting was facilitated by the panel in order to assess how well the translated version was understood. Their comments were recorded and the translation was modified. Then another bilingual medical researcher independently back-translated the finalized Chinese version into English. The panel compared the original English version with the back-translated Chinese version, and revised the suboptimal translated words/phrases to ensure consistency with Chinese culture. For example, Item 21 refers to *financial difficulties related to HIV/AIDS* such as *problems with superannuation payouts*. However, the social security system in China does not provide retirement protection plans for most unemployed people and, thus, the item was modified as follows, *problems with daily necessity payments* (in Chinese*)*. The final Chinese version of SS-HIV was used in this study. Please see details of CSS-HIV in S1 Appendix.

#### Chinese Patient Health Questionnaire-9 (PHQ-9)

Depression was measured by the PHQ-9, a 9-item screening instrument based on diagnostic criteria for major depression from the *Diagnostic and Statistical Manual of Mental Disorder*, *Fourth Edition* (DSM-IV) [33, 34]. Each item is rated on a 4-point scale ranging from 0 (none of the time) to 3 (almost every day). Cut-off scores of 5, 10 and 15 represent mild, moderate and severe levels of depression, respectively [33]. The PHQ-9 has shown good reliability and validity in various Chinese samples including HIV-infected individuals [35, 36].

#### Chinese Generalized Anxiety Disorder Scale (GAD-7)

GAD-7 is a screening instrument for anxiety, which consists of 7 items that reflect the symptom criteria for general anxiety disorder from the DSM-IV [37, 38]. Respondents rate each item on a 4-point Likert scale regarding how often they have been bothered by an anxiety symptom in the last two weeks. Higher scores indicate higher levels of anxiety symptoms. A score over 5 was used to indicate a “mild-severe” level of anxiety, similar to levels of depression on the PHQ-9 [38]. The GAD-7 has been utilized in various Chinese samples including HIV-positive [35, 36], showing good reliability and validity.

### Statistical analysis

Firstly, we conducted a confirmatory factor analysis (CFA) to examine the three-factor structures proposed by Pakenham and Rinaldis [22]. The following cutoff criteria for the goodness of fit indices were used: χ^*2*^/*df* <3, Comparative Fit Index (CFI)>0.90, Goodness of Fit Index (GFI)>0.90, Tucker-Lewis Index (TLI)>0.90, Root Mean Square Error of Approximation (RMSEA) <0.05 [39]. The results from the CFA were not satisfied (see Table 1), so we conducted an exploratory factor analysis (EFA) to explore the underlying factor structure of the 23 items. The sample adequacy was assessed by the Kaiser-Meyer-Olkin (KMO) test and Bartlet’s test. Varimax rotation method was used in the EFA. When the model fit was inadequate, we identified problematic items. Items were retained if they met the following criteria: 1) correlation coefficients with the whole scale higher than 0.40; 2) factor loadings greater than 0.40 on only one factor [40]. After removal of problematic items, we conducted EFA and CFA again on the retained items. Table 1 shows model fit indices of the original and revised versions of the CSS-HIV. The model with the best model fit was retained.

**Table 1:** Goodness of fit indices of the CFA models on the CSS-HIV.

Model description	χ^*2*^/*df*	CFI	GFI	TLI	IFI	RMSEA
Original (SS-HIV) 23-item 3-factor	5.480	0.854	0.842	0.837	0.855	0.082
CSS-HIV 23-item 3-factor	4.395	0.889	0.881	0.877	0.890	0.071
CSS-HIV 22-item 3-factor	4.268	0.902	0.892	0.890	0.902	0.070
CSS-HIV 17-item 3-factor	3.982	0.923	0.924	0.909	0.923	0.067

Notes: The following cutoff criteria for the goodness of fit indices were used: χ^*2*^/*df* <3, Comparative Fit Index (CFI)>0.90, Goodness of Fit Index (GFI)>0.90, Tucker-Lewis Index (TLI)>0.90, Root Mean Square Error of Approximation (RMSEA) <0.05.

Internal reliability and test-retest reliability was assessed using the criterion of Cronbach’s alpha ≥0.70 and the intra-class correlation coefficient (ICC) ≥0.70, respectively [39, 41]. Inter-correlations among the three factors were also calculated using Spearman correlation coefficients. Concurrent validity was evaluated by correlations between the CSS-HIV and symptoms of depression and anxiety.

The CFA analyses were performed with AMOS 7.0 (SPSS Inc., Chicago, IL, USA). All the other analyses were performed with SPSS version 17.0 (SPSS Inc., Chicago, IL, USA). Significance level of this study was set at 0.05.

## Results

### Participants’ characteristics

Of the 667 participants, 7.3% were female, and the mean age was 31.9 years with a range of 18 to 76 years (SD = 10.6). Most were single (62.7%), had attained tertiary education (46.3%), and had a stable job (56.2%). Most respondents (83.5%) were newly diagnosed (less than 1 month), and infected through sexual transmission (95.2%), in particular homosexual transmission (57.1%). Over one-third (36.7%) had HIV-related clinical symptoms, while 12.6% had a CD4 count lower than 200/μl.

### Factor analyses

First, we conducted CFA on the original 23-item three-factor model, and poor levels of goodness of fit were found (Table 1). Then we conducted EFA with Varimax rotation on the 23 items. The KMO value was 0.950, and the Bartlett’s test of Sphericity was statistically significant (^*2*^ = 7126.713, P = 0.000), indicating the adequacy of proceeding with factor analysis. The EFA results indicated a three-factor model, but some items loaded on different factors rather than the original one. We conducted CFA on the revised 23-item three-factor model, which also showed a poor fit to the data with high χ^*2*^/*df*, CFI, GFI and TLI below 0.90, and RMSEA over 0.7.

Then we removed Item 10 (*increased drug/alcohol use*) which was correlated with the total score <0.4 (*r* = 0.199, *p* = 0.000), and 85.5% endorsed “not at all”. EFA (with Varimax) and CFA were carried out on the retained 22 items, but the results still showed that the 22-item three-factor model was not ideal (Table 1).

Next, we removed 5 items (Items 6, 7, 12, 16, 22) with double loadings (>0.4) based on the EFA on 22 items, and conducted EFA (with Varimax) and CFA on the 17 items. The EFA yielded a three-factor solution that accounted for 56.274% of the variance, and the CFA goodness-of-fit measures supported the adequacy of this solution (CFI, GFI, TLI and IFI >0.90). Therefore, the 17-item three-factor structure was retained as the final model.

Based on the EFA results of the 17-item CSS-HIV, Factor 1 consisted of 6 items representing “social stress” (Items 4, 5, 11, 13, 14, 23) and accounted for 40.532% of the variance; Factor 2 consisted of 6 items representing “emotional stress” (Items 1, 2, 3, 8, 9, 15) and accounted for 8.687% of the variance; whereas Factor 3 consisted of 5 items representing “instrumental stress” (Items 17, 18, 19, 20, 21) and accounted for 7.054% of the variance. Eleven out of 17 items fell into the original SS-HIV factors, and item loadings ranged from 0.471 to 0.765 (Table 2).

**Table 2:** Rotated factor matrix for the 17-item CSS-HIV.

Sub-scales and item description	Mean (SD)	Factor 1	Factor 2	Factor 3
***Social stress***	10.94(6.01)			
04. Confidentiality/privacy concerns related to HIV/AIDS	2.46(1.43)	0.765		
14. Difficulties in telling others of your HIV/AIDS status	1.91(1.45)	0.708		
13. Overly attentive to bodily functions or changes	1.71(1.30)	0.695		
11. Discrimination/stigma concerns related to HIV/AIDS	1.74(1.46)	0.668		
23. Reducing risk of infection	1.88(1.38)	0.640		
05. Sexual difficulties related to HIV/AIDS	1.23(1.27)	0.471		
***Emotional stress***	6.12(5.07)			
02. Relationship difficulties related to HIV/AIDS	0.75(1.02)		0.741	
01. Distressing emotions related to HIV/AIDS	1.49(1.14)		0.677	
09. Suicidal thoughts/attempts related to HIV/AIDS	0.60(1.00)		0.658	
15. Boredom related to HIV/AIDS	1.03(1.14)		0.644	
08. Isolation related to HIV/AIDS	1.15(0.94)		0.632	
03. Grief/bereavement related to HIV/AIDS	1.30(1.26)		0.626	
***Instrumental stress***	4.60(4.28)			
18. Difficulty with health care system	0.88(1.17)			0.746
19. Difficulty with treatment related to HIV/AIDS	1.05(1.23)			0.720
21. Financial difficulties related to HIV/AIDS	0.81(1.11)			0.718
20. Transport difficulties related to HIV/AIDS	0.49(0.90)			0.657
17. Employment difficulties related to HIV/AIDS	1.35(1.34)			0.526
**The total score of CSS-HIV**	21.65(13.22)			
Eigenvalue		6.891	1.477	1.199
Proportion of explained variance (%)		40.532	8.687	7.054

### Reliability

Cronbach’s α for the 17-item CSS-HIV was 0.906 for the whole scale. As displayed in Table 3, internal reliability coefficients of subscales ranged from 0.791 to 0.846; the inter-correlations among the three subscales were moderate, ranging from 0.567 to 0.656. The ICC was 0.908 for the whole scale and ranged between 0.766 and 0.829 for the subscales.

**Table 3:** The interclass correlations, internal reliability and test-rest reliability of 17-item CSS-HIV factors.

Factors	Factors			Cronbach’s α	ICC
	Social stress	Emotional stress	Instrumental stress		
Social stress	-	-	-	0.818	0.766
Emotional stress	0.656*	-	-	0.846	0.823
Instrumental stress	0.567*	0.635*	-	0.791	0.829

Note: Internal reliability and test-retest reliability was assessed using the criterion of Cronbach’s alpha ≥0.70 and the intra-class correlation coefficient (ICC) ≥0.70.

* *p* <0.05

### Concurrent validity

As shown in Table 4, people with more HIV-related symptoms were more likely to report higher CSS-HIV total and subscale scores. Although the correlations were weak, younger age was associated with greater social stress (*r* = -0.104); and people infected through heterosexual transition tended to have lower stress than those infected through homosexual and other modes, especially emotional stress (*r* = 0.080). Gender, education and CD4 counts were unrelated to the CSS-HIV total and factor scores.

**Table 4:** Correlation of the 17-item CSS-HIV to demographics and validating scales.

	CSS-HIV	Social stress	Emotional stress	Instrumental stress
Gender (female)	-0.011	-0.034	0.014	-0.001
Age	-0.059	-0.104**	-0.038	0.005
Education	0.049	0.071	-0.042	0.015
Transmission mode	0.088*	0.068	0.080*	0.069
Numbers of symptoms	0.248**	0.200**	0.236**	0.243**
CD4 count	-0.006	0.067	0.013	-0.050
Depression (PHQ-9)	0.661**	0.531**	0.688**	0.518**
Anxiety (GAD-7)	0.678**	0.561**	0.711**	0.510**

Note. Education was coded as: 0 = primary education and below, 1 = secondary education, and 2 = tertiary education; transmission mode were coded as: 0 = heterosexual transmission, 1 = homosexual transmission, and 2 = others.

* *p* <0.05

** *p* <0.01

In terms of depression and anxiety, the mean score of PHQ-9 was 78.09±6.63, and ranged from 0 to 27. 63.1% had depression symptoms (≥5) and 34.5% were classified in “moderate-severe” depression range (≥10). The mean GAD-7 score was 6.69±5.77, with a range from 0 to 21. The prevalence of anxiety was 58.6% (≥5), and 27.3% were classified with “moderate-severe” anxiety symptoms (≥10).

The Spearman correlation analysis indicated that the total stress score, as well as the three subscales, were significantly and positively correlated with both depression and anxiety. The correlations were moderate, ranging from 0.510 to 0.711.

## Discussion

This study presents the psychometric characteristics of a Chinese version of the HIV/AIDS Stress Scale (SS-HIV) in a representative sample of people living with HIV in central China. The results suggested that the 17-item CSS-HIV with three factors has adequate internal reliability, test-retest reliability and concurrent validity.

Item 10 which referred to substance abuse related to HIV/AIDS was removed. We found that most of the participants (86.7%) reported no increased drug/alcohol intake related to HIV/AIDS, although some prior research has shown that life stress is associated with increased substance use in people living with HIV [10, 42]. Meade’s study also found that substance abuse was the least commonly endorsed item among the 23 items [27]. It may indicate a cultural discrepancy in how Western and Chinese individuals react to stressful events. Additionally, other problematic items tended to be related to the original emotional/existential stress dimension including: *concerns about death related to HIV/AIDS*, *difficulties in coming to terms with HIV/AIDS status*, *difficulty dealing with HIV-related symptoms or illness*, *and religious/existential difficulties related to HIV/AIDS*. The fact that these items did not load unambiguously on the original emotional/existential stress dimension may reflect cultural differences in perceptions of these stressor and marked progress in the treatment and management of HIV/AIDS over the past 10 years. Regarding the latter, these stressors (particularly the existential stressors) are less likely to be prominent in the context of current relatively effective medical treatments, which have changed HIV/AIDS from being a life threatening illness to a chronic health condition.

Findings from the present study confirm the three-dimensional structure of HIV/AIDS related stress: social, emotional, and instrumental stress. In the 17-item three-factor structure of CSS-HIV, the majority of items loaded onto these original three factors. However, cultural differences in how some of the HIV/AIDS stressors in the CSS-HIV are experienced emerged. There were six items that loaded onto other factors rather than the original one, and the changes were mostly between social stress and emotional stress. According to Lazarus and Folkman’s (1984) conceptualization of stress and coping [23], the extent to which an event is perceived as stressful in large part depends on the person’s appraisal of the event with respect to its potential for harm, controllability and mastery. These appraisals are in turn shaped by cultural and social contexts [43]. There is considerable conceptual overlap between social stress and emotional stress as demonstrated by the moderately high correlation between these two factors in the present study (*r* = 0.656). Different cultural contexts may alter the poignancy of the emotional or social elements of stressors represented in the items that changed between the social and emotional stress factors.

Take the item *Suicidal thoughts/attempts related to HIV/AIDS* as example. This item originally loaded on the social stress factor, but in the present study it loaded on the emotional stress factor. There are various motivations for suicide. For instance, the interpersonal theory of suicide suggests that perceived burdensomeness and social alienation are the key elements that arouse one’s desire for death [44]. In Shneidman’s model of suicide [45], a desire to end unendurable psychological pain/psychache is considered to be the core feature of suicide. In China, intrapersonal motivations (e.g. feeling depressed, hopelessness, and a desire to escape of pain,) were endorsed more frequently than the interpersonal factors among people living with HIV who reported suicide ideation/attempts [46–48]. These findings are consistent with the suicide thoughts/attempts item loading on the emotional stress factor in the present study.

The reliability findings supported that the 17-item CSS-HIV and its three subscales have good internal consistency (Cronbach’s α 0.791–0.906), which were similar to findings of the original 23-item scale (0.76–0.85), but better than the 23-item Chinese versions developed by Lin et al. (0.55–0.73) [29] and Gao et al. (0.55–0.75) [30]. The ICCs of the whole scale and subscales ranged from 0.766 to 0.908, which indicates that the 17-item CSS-HIV has acceptable test-retest reliability over the short-term (4 weeks).

Consistent with Pakenham and Rinaldis’ study [22], this study also showed that problems related to social stress were most troublesome, and people with more HIV-related symptoms were likely to have higher stress (social, instrumental and emotional/existential stress). Previous studies conducted by Lin et al [29] and Gao et al [30], have shown that the SS-HIV has concurrent validity with a number of other measures including Beck Depression Inventory (BDI) [29], Center for Epidemiological Studies Depression Scale (CES-D) [30] and Self-rating Anxiety Scale (SAS) [29, 30] among Chinese HIV-infected people. In the current study, HIV-related stress was also found to be significantly and moderately positively correlated with depression and anxiety (*r* = 0.661 for PHQ-9 and 0.678 for GAD-7; p<0.001) and, thus, the construct validity of this 17-item scale was supported, and demonstrates the expected link between HIV/AIDS related stress and depression and anxiety.

The main limitation of this study is the sample characteristics. Most of the participants were male and more than half of them were infected via homosexual transmission. HIV-infected sub-populations, such as injecting drug users (IDU), female sex workers and pregnant women may have different stressful experiences due to HIV-infection. For example, Item 10 refers to *increased drug/alcohol intake*, which might be a prominent stressor for HIV-infected IDUs, was the least stressful event in our sample where only 1.2% were IDUs. Another limitation is the lack of representation of people with longer time of infection and advanced courses of AIDS, and this may have produced a bias towards lower levels of stress. Future research on the CSS-HIV should be conducted with other Chinese HIV-infected groups, and continue to evaluate its psychometric properties.

In conclusion, results from the present study show that the 17-item CSS-HIV is a psychometrically sound instrument for measuring HIV/AIDS-related stress among people living with HIV in China. The CSS-HIV shows potential in identifying people living with HIV who may be at risk for mental health problems. The CSS-HIV is also useful in identifying important stressors among HIV-infected Chinese, which will help to inform the development of targeted stress management programs. Having a common scale that assesses HIV-related stress will also assist research that explores comparisons in HIV across countries.

## Supporting Information

S1 Appendix The English and Chinese versions of SS-HIV. (PDF)
