# Supplementary material for: Cross-Culture Validation of the HIV/AIDS Stress Scale: The Development of a Revised Chinese Version
Source: PLoS One. 2016 Apr 4;11(4):e0152990. doi: 10.1371/journal.pone.0152990 (PMC4820124; doi:10.1371/journal.pone.0152990)
Supplement: S1 Appendix — (PDF) [file pone.0152990.s001.pdf]

HIV/AIDS STRESS SCALE

Below is a list of problems that people who have HIV/AIDS sometimes have. For each problem category there are two examples which further describe the problem. **Your own** examples may be somewhat **different** from the ones provided, so long as they seem to you to be examples of the problem category you are being asked to rate. In order to endorse a problem it must be in someway be related to HIV. That is, the problem must at least in part be caused or by or be exacerbated by being HIV infected. Please read each one carefully. After you have done so, please cross one of the numbers to the right that best describes HOW TROUBLESOME THAT PROBLEM HAS BEEN FOR YOU DURING THE PAST MONTH INCLUDING TODAY. Cross only one number for each problem and do not skip any items. If you change your mind erase your first mark carefully.

**HOW MUCH WERE YOU TROUBLED BY:**	**Not At All**	**A Litter Bit**	**Moder-ately**	**Quite A Bit**	**Extre-mely**
1. **Distressing emotions related to HIV/AIDS** (e.g., you feel angry or fearful; you feel anxious or depressed)	0	1	2	3	4
2. **Relationship difficulties related to HIV/AIDS** (e.g., you have arguments with your support person about how to best care for your health; you have difficulty establishing a relationship)	0	1	2	3	4
3. **Grief/bereavement related to HIV/AIDS** (e.g., you are concerned about your own losses such as loss of independence; you are grieving for the loss of a loved one from AIDS)	0	1	2	3	4
4. **Confidentiality/privacy concerns related to HI** (e.g., you are concerned about your HIV status breached; you are reluctant to tell other of your HIV/AIDS status)	0	1	2	3	4
5. **Sexual difficulties related to HIV/AIDS** (e.g., you're finding it hard to maintain safe sex behaviours; you are sexually frustrated)	0	1	2	3	4
6. **Difficulties in coming to terms with your HIV/A** (e.g., you can't accept that you have HIV/AIDS; you refuse to even think about your HIV/AIDS)	0	1	2	3	4
7. **Concerns about death related to HIV/AIDS** (e.g., you are preoccupied with dying; you don't think about the possibility that you may die from HIV/AIDS)	0	1	2	3	4

8. **Isolation related to HIV/AIDS** (e.g., you have less contact with others because of your HIV/AIDS; you don't get invited out much now that you have HIV/AIDS)	0	1	2	3	4
9. **Suicidal thoughts/attempts related to HIV/AID[illegible]** (e.g., you have thoughts of ending your life; you have actually attempted to end your life)	0	1	2	3	4
10. **Increased drug/alcohol intake related to HIV/[illegible]** (e.g., you use drugs and/or alcohol more now: you are often "high" or "drunk"	0	1	2	3	4
11. **Discrimination/stigma concerns related to HI[illegible]** (e.g., you are concerned that you will be discriminated against because of your HIV/AIDS; you feel as if you have not been treated with respect)	0	1	2	3	4
12. **Religious/existential difficulties related to HIV** (e.g., you are having difficulty searching for meaning in your life; you are struggling to make sense of the predicament you are in)	0	1	2	3	4
13. **Overly attentive to bodily functions or change[illegible]** (e.g., you are constantly on the watch out for HIV-related symptoms; you are overly attentive to any new physical changes such as appearance of a rash)	0	1	2	3	4
14. **Difficulties in telling other of your HIV/AIDS [illegible]** (e.g., you don't know who, how or when to tell of your HIV status; you have told only one or two people of your HIV status)	0	1	2	3	4
15. **Boredom related to HIV/AIDS** (e.g., you are unable to use your free time doing things you would normally enjoy; you often find yourself sitting about doing nothing)	0	1	2	3	4
16. **Difficulty dealing with HIV-related symptoms** (e.g., you often have difficulty dealing with fatigue or nausea; you have pain and physical discomfort most of the time)	0	1	2	3	4
17. **Employment difficulties related to HIV/AIDS** (e.g. you can't obtain/maintain employment because of illness; you are concerned about work-related stress)	0	1	2	3	4

18. **Difficulty with health care system** (e.g., you have difficulties in getting access to health services such as dentists or home care)	0	1	2	3	4
19. **Difficulties with treatment related to HIV/AID** (e.g. you have difficulties managing side effects from treatments; you can't decide on a treatment approach)	0	1	2	3	4
20. **Transport difficulties related to HIV/AIDS** (e.g., you have difficulty getting appropriate transport to places; public transport is physically demanding)	0	1	2	3	4
21. **Financial difficulties related to HIV/AIDS** (e.g. you are unable to pay debts; you have problems with superannuation payouts)	0	1	2	3	4
22. **Planning difficulties related to HIV/AIDS** (e.g. uncertainty with your health makes career planning difficult; you don't know whether to start new projects)	0	1	2	3	4
23. **Reducing risk of infection** (e.g. you are preoccupied with thoughts about infecting others with HIV; you are concerned that some of the behaviours you engage in may infect others)	0	1	2	3	4

HIV/AIDS 压力量表

指导语：以下是 HIV 感染者和 AIDS 患者常会遇到的一些问题。每一类别的问题后都举了两个例子对其进行描述。您自己的例子可能与之有所差别，但只要是与这一类问题相关的即可，所有的问题都与 HIV 有关，或者至少一定程度上是因 HIV 感染而导致或因 HIV 感染而加重。请您逐条阅读每一个条目，并根据您的情况在合适的选项数字上划圈。

**过去 1 个月内（包括今天），这个问题多大程度上困扰了您？**	**完全没有**	**有一点**	**中等**	**相当大**	**特别大**
1.与 HIV/AIDS 有关的悲伤情绪（例如，您感到愤怒或害怕；您感到焦虑或沮丧）	0	1	2	3	4
2.与 HIV/AIDS 有关的人际关系困难（例如，您和亲人就如何更好照顾您的健康有过争吵；难以和别人建立联系）	0	1	2	3	4
3.与 HIV/AIDS 有关的悲哀或丧亡（例如，您担心自己的各种损失，如失去自立能力等；因所爱的人死于 AIDS 而悲伤）	0	1	2	3	4
4.关注与 HIV/AIDS 有关的保密性或隐私(例如，您担心自己的 HIV 状况被别人知道；您不愿意将自己的 HIV/AIDS 状告诉他人）	0	1	2	3	4
5.与 HIV/AIDS 有关的性(如与伴侣间的性生活)困难（例如，您发现难以维持安全的性生活；您在性生活方面有困难）	0	1	2	3	4
6.由您的 HIV/AIDS 状况引起的有关困难（例如，不能接受自己患有艾滋病；甚至不愿去考虑自己的 HIV/AIDS）	0	1	2	3	4
7.与 HIV/AIDS 有关的死亡(例如，您脑子里总有死亡的念头；您不去想自己死于 HIV/AIDS 的可能性）	0	1	2	3	4
8.与 HIV/AIDS 有关的隔离（例如，因为 HIV/AIDS，您与别人的接触减少；由于患有 HIV/AIDS，别人很少邀请您外出）	0	1	2	3	4
9.与 HIV/AIDS 有关的自杀想法（或自杀未遂）与 HIV/AIDS 有关的自杀的想法/行为（例如，您有结束自己生命的想法；实际上，您曾尝试过结束自己的生命）	0	1	2	3	4
10.因为 HIV/AIDS，增加了毒品和酒精的使用（例如，您现在更多地吸毒和（或）喝酒；您经常喝高或喝醉）	0	1	2	3	4
11.担心与 HIV/AIDS 有关的歧视或侮辱（例如，您很担心因为 HIV/AIDS 受歧视；您感觉没有受到别人的尊重）	0	1	2	3	4
12.与 HIV/AIDS 有关的宗教信仰或人生意义的困难（例如，您很难找到生命的意义；您还在努力理解自己的困难处境）	0	1	2	3	4

13.过分关注身体功能或身体的变化（例如，您时刻关注与HIV 有关的症状；您过分关注身体上所有新的变化，例如一个皮疹的出现）	0	1	2	3	4
14.难以告诉他人自己的 HIV/AIDS 状况（例如，您不知道何时、向何人、以何种方式告知自己的 HIV 情况；您仅把自己的 HIV/AIDS 情况告诉给一两个人）	0	1	2	3	4
15.与 HIV/AIDS 有关的厌烦（例如，您无法利用空闲时间做自己平时喜欢做的事情；您经常发现自己无所事事地坐着）	0	1	2	3	4
16.处理与 HIV/AIDS 有关的症状或疾病感受上的困难(例如，您经常难以应付疲劳或恶心；您总感到疼痛和身体不适）	0	1	2	3	4
17.与 HIV/AIDS 有关的工作方面的困难（例如，因为患病，您不能找到或维持工作；您担心工作相关压力）	0	1	2	3	4
18.在医疗卫生服务体系方面的困难（例如，您难以获得医疗服务，例如牙医的服务或家庭护理）	0	1	2	3	4
19.与 HIV/AIDS 有关的治疗方面的困难（例如，您难以处理治疗带来的副作用；您不能决定一个治疗方案）	0	1	2	3	4
20.与 HIV/AIDS 有关的交通困难（例如，您难乘坐合适的交通工具去不同的地方；乘坐公共交通车让您感到体力不支）	0	1	2	3	4
21.与 HIV/AIDS 有关的经济困难（例如，您难以偿还债务；您买不起生活必需品）	0	1	2	3	4
22.与 HIV/AIDS 有关的规划生活的困难（例如，对健康的不确定使您难以做出职业规划；不知是否应该开展新的工作计划）	0	1	2	3	4
23.降低感染风险（例如，您总担心将 HIV 传染给他人；您担心自己的某些行为会把病毒传染他人）	0	1	2	3	4
